# Characterisation, Flocculation Efficiencies and Mechanisms of Bioflocculants Derived from *Klebsiella pneumoniae* and *Meyerozyma guilliermondii*

**DOI:** 10.3390/polym17233155

**Published:** 2025-11-27

**Authors:** Mathari Boshomane, Kgabo Moganedi, Tsolanku Sidney Maliehe, Cyril Tlou Selepe, Nkoana Ishmael Mongalo, Tlou Nelson Selepe

**Affiliations:** 1 Department of Biochemistry, Microbiology and Biotechnology, University of Limpopo, Private Bag X1106, Polokwane 0727, South Africa; kgabo.moganedi@ul.ac.za; 2 Department of Biochemistry, Genetic and Microbiology, University of KwaZulu Natal, Private Bag X 54001, Durban 4000, South Africa; maliehet@ukzn.ac.za; 3 Department of Chemical Sciences, University of Johannesburg, Private Bag XX61, Johannesburg 2006, South Africa; cyrilkatlego15@gmail.com; 4 College of Agriculture and Environmental Science (CAES), University of South Africa, Private Bag X06, Florida 0710, South Africa; mongani@unisa.ac.za; 5 Department of Water and Sanitation, University of Limpopo, Private Bag X1106, Polokwane 0727, South Africa; tlou.selepe@ul.ac.za

**Keywords:** *Klebsiella pneumoniae*, *Meyerozyma guilliermondii*, flocculation activity, flocculation mechanism, wastewater treatment

## Abstract

Evaluation of characteristics and flocculation mechanisms of microbial flocculants facilitates the identification of potential applications and informs the fine-tuning of operational conditions for maximum activity. Therefore, this study aimed to characterise and optimise the operational conditions of bioflocculants produced from *Klebsiella pneumoniae* and *Meyerozyma guilliermondii* for potent wastewater treatment. Scanning electron microscopy, X-ray Diffraction (XRD), Thermogravimetric Analysis (TGA) and Fourier Transform Infrared Spectroscopy (FTIR) were employed to assess the surface morphology, crystalline structure, thermal stability, and functional group composition of the bioflocculants. Their cytotoxicity was assessed using the tetrazolium bromide-based assay against human colorectal adenocarcinoma (CaCO-2) cell lines. Flocculation efficiencies and mechanisms were determined using Jar and zeta potential assays, respectively. The bioflocculant from *K. pneumoniae* (Kp1) revealed a fibrous morphology, whereas that from *M. guilliermondii* (Mg1) displayed a granular structure. FTIR spectra revealed hydroxyl, amine, and alkene groups as key functional groups, while TGA analysis indicated that Kp1 was thermally unstable, contrary to Mg1, which exhibited good thermal stability. Bioflocculants Kp1 and Mg1 exhibited COD removal of 90.86% and 93.12% and turbidity reductions of 92.65% and 92.74%, respectively. Zeta potential analysis revealed that bioflocculant Kp1 primarily flocculated through charge neutralisation, while Mg1 employed a bridging mechanism. These bioflocculants illustrated strong potential to treat wastewater. However, the observed cytotoxic effect at increased concentrations warrants cautious handling and application in lower doses.

## 1. Introduction

Water pollution continues to pose major threats to global health and environmental sustainability [1]. With the growing demand for safe and reusable water, efficient wastewater treatment has become an essential strategy for addressing pollution [2]. Flocculation is one of the physicochemical methods widely employed for wastewater treatment. It involves the aggregation of destabilised colloidal pollutants into larger flocs through the action of flocculants. Chemical flocculants are commonly used in wastewater treatment because of their cost-effectiveness and high efficiency [3]. However, concerns over their toxicity, non-biodegradability, and residual contamination have shifted attention toward bioflocculants, which are considered safer and more eco-friendly alternatives [4].

Microbial bioflocculants are complex biopolymers predominantly composed of polysaccharides, proteins, glycoproteins, or nucleic acids [5]. They have attracted significant attention as environmentally friendly alternatives to conventional chemical flocculants as they are biodegradable and non-toxic and produce no secondary pollution [6]. Their effectiveness arises from their diverse molecular structures and functional groups, which influence the physicochemical interactions that occur during the flocculation process [4]. Their performance is strongly influenced by operational parameters such as dosage, pH, temperature, agitation, cation concentration, and contact time [7]. Thus, proper optimisation of these factors is essential to achieving high flocculation efficiency and process consistency [8]. In this regard, statistical optimisation approaches such as Response Surface Methodology (RSM) have proven effective in analysing multivariable interactions and identifying optimal conditions with minimal experimental trials [9,10].

The flocculation process involves complex physicochemical interactions between flocculants and suspended pollutants. The dominant mechanisms are generally classified into charge neutralisation and polymer bridging [11]. Charge neutralisation occurs when oppositely charged flocculants neutralise the surface charge of colloidal pollutants, reducing electrostatic repulsion and enabling aggregation [12]. Polymer bridging, on the other hand, involves long-chain polymeric flocculants adsorbing onto multiple pollutants simultaneously, binding them together into larger flocs [13]. The relative contribution of each mechanism depends on factors such as bioflocculant molecular structure, charge density, and solution chemistry (e.g., pH and ionic strength). While the mechanisms of chemical flocculants such as aluminium and iron salts are well characterised [14,15], the mechanistic understanding of microbial bioflocculants remains limited [16]. This knowledge gap restricts their broader industrial application, as uncertainties in mechanism hinder optimisation, scale-up, and cost-effective process integration [17]. Consequently, elucidating the operational and mechanistic behaviour of microbial bioflocculants is essential for enhancing their reliability and applicability in real wastewater systems.

In our previous study, two bioflocculant-producing strains (*Klebsiella pneumoniae* PQ177892 and *Meyerozyma guilliermondii* PQ305552) were isolated from municipal wastewater effluent. The isolates were subsequently optimised for enhanced bioflocculant production, yielding glycoprotein bioflocculants, which were then designated as Kp1 and Mg1 for *K. pneumoniae* and *M. guilliermondii*, respectively. The production yields were 8.60 g/L for Kp1 and 5.20 g/L for Mg1 (unpublished data). The present study aimed to characterise the produced bioflocculants and to refine their operational conditions. Moreover, we predicted the underlying flocculation mechanisms of the bioflocculants in wastewater treatment.

## 2. Materials and Methods

### 2.1. Characterisation of the Previously Extracted Bioflocculants

#### 2.1.1. Morphology and Elemental Composition of the Bioflocculants

The bioflocculants were prepared by rinsing them twice in phosphate buffer saline (0.01 M, pH 7.4) and fixing them in 3 mL of 3% buffered glutaraldehyde overnight. Afterwards, they were dehydrated for ten minutes at 4 °C using ethanol grades of 20, 50, 80 and 100%, respectively. The bioflocculants underwent a critical point CO_2_ drying process and were subsequently mounted on aluminium stubs. They were sputter-coated with gold (90 Å thick) for 30 min in polaron Sc 7640 (Carl Zeiss, Oberkochen, Germany). Thereafter, the scanning was conducted and visualized at different magnifications with the scan electron microscope (SEM) (Leo Electron Microscopy Ltd., Cambridge, UK) [18]. The quantitative elemental composition of the bioflocculants was determined using the in-built EDX spectrometry at 20 kV on a Dx4 Prime EDX spectrometer (Bruker, Billerica, MA, USA) fitted with an X-flash detector on SEM [19].

#### 2.1.2. Functional Groups of the Bioflocculants

FTIR spectroscopic analysis was performed using the Bruker Alpha 1 Spectrometer to evaluate the active groups responsible for bioflocculation. The spectrophotometer and its resolution were set to the desired scan ranges of 4000–400 cm^−1^ and 4–8 cm^−1^, respectively. Thereafter, the background spectrum was collected by scanning the empty sample holder before collecting the sample spectrum. The samples were placed in the sample holder, properly aligned and their spectrum was collected. The scan was then repeated 3 times to ensure spectral reproducibility and the background spectrum was subtracted from the ample spectrum to obtain the accurate spectrum. Afterwards, the functional groups of the bioflocculants were assigned to the absorption bands using a spectral library [20].

#### 2.1.3. Crystallinity of the Bioflocculants

The crystallinity profiles of the bioflocculants were evaluated using the XRD analysis method as denoted by Shah et al. [21]. The XRD instrument (Malvern Panalytical, Almelo, Netherlands) was set to the desired scan range (5–80° 2θ), while the scan speed and step size were set at (0.5–1° 2θ/minute). Thereafter, the appropriate radiation (Cu Kα) was selected. The dried bioflocculant samples were ground using a pestle and mortar and sieved to give a uniform particle size. Afterwards, bioflocculants were loaded evenly and pressed firmly on the sample holder. The sample holder was inserted in the XRD instrument, and the diffraction pattern was collected by scanning the sample over the specified 2θ range. The background signal from the diffraction pattern was subtracted and the peaks of diffraction were identified manually. Following that, the peaks were indexed to determine crystal structure and lattice parameters. The crystallinity and crystal size were calculated using manual analysis.

#### 2.1.4. Pyrolysis Property of the Bioflocculants

The thermogravimetric mass 2 was used to study the thermal degradation behaviour of the bioflocculants during pyrolysis following the method described by Nwodo et al. [22]. In brief, the thermogravimetric analyser (TGA) was operated within a temperature range of 25–1000 °C at a controlled heating rate of 10–20 °C/minute under an inert nitrogen atmosphere flowing at 40 mL/minute to prevent oxidative degradation. Approximately 10 mg of dry, powdered bioflocculant sample was placed in the TGA, and the weight loss as a function of temperature was continuously recorded. Thereafter, the pyrolysis parameters (onset temperature, peak temperature and residual weight) were calculated.

#### 2.1.5. Biosafety Assessment of the Purified Bioflocculants

The biosafety of the bioflocculants was assessed by determining their cytotoxic effects on human colorectal adenocarcinoma cells (CaCO-2) using the 3-(4,5-dimethylthiazol-2-yl)-2,5-diphenyl tetrazolium bromide assay [23]. The mixture of complete culture media (CCM), minimal essential medium (MEM) supplemented with 5% foetal calf serum and 0.1% gentamicin was used to grow the cells to 80% confluence in 25 cm^3^ flasks before they were harvested. The cells were re-suspended in growth media at a concentration of 5 × 10^4^ cells mL^−1^, and a 96-well plate was used to incubate them for 24 h at 37 °C in 5% CO_2_. Following the removal of the CCM, the cells were exposed to varying doses of bioflocculants (50–200 µg/µL). Doxorubicin was used as the positive control and 0.1% dimethyl sulfoxide (DMSO) was used as the negative control. The cells underwent a 24-h re-incubation at 37 °C in 5% CO_2_. Following incubation, the medium was discarded and replaced with 100 µL of newly prepared CCM. Subsequently, the MTT solution was aspirated from the wells, and the formazan particles were dissolved in 50 µL of DMSO. The OD of the samples was measured at 540 nm using a microplate reader (Varioskan Flash 3001, Thermo Fisher Scientific, Vantaa, Finland) to determine the MTT reduction. Percentage cell inhibition (%CI) was evaluated using the formula:

%CI = (A_o_ − A)/A × 100, (1)

where A_o_ and A represent the OD readings of untreated samples and treated samples, respectively.

### 2.2. Optimisation of Operational Conditions of the Extracted Bioflocculants

#### 2.2.1. Effect of Cations on Flocculation

The impact of different cations on percentage flocculating activity (%FA) was analysed using the one-factor-at-a-time (OFAT) approach, with other variables such as dosage, and shaking time held constant. Briefly, 3 mL of cations (calcium chloride (CaCl_2_), potassium chloride (KCl), sodium chloride (NaCl), aluminium chloride (AlCl_3_), and ferric chloride (FeCl_3_)) were separately poured into kaolin solution (95 mL) and bioflocculant solution (2 mL). The mixtures were vigorously shaken at 200 rpm for 1 min. Afterwards, a gentle stirring of the mixture was performed at 40 rpm for 2 min and the mixture was then allowed to settle at room temperature for 5 min. A control was prepared similarly, replacing the bioflocculant with sterile distilled water. The optical density (OD) of the upper phase of the mixture was measured at 550 nm using a Genesis 10 UV scanning spectrophotometer (Thermoscientific). Subsequently, the %FA was calculated using Equation (2) [24].

%FA = [(A − B)]/A × 100,(2)

whereby, A represents the OD of the control and B represents the OD measurement of the sample.

#### 2.2.2. Effect of Operational Parameters on Flocculation

RSM using Box Behnken Design (BBD) was employed to optimise the experimental variables such as cation, dosage, pH and shaking time to achieve the maximum flocculating activity. Each factor was studied at three levels, and a total of 27 experimental runs were performed. The experimental model was developed using Minitab 21.2 (trial version). The measured data was fitted into a second-degree polynomial model, which was expressed as:

(3) $$Y=\beta_{0}+\sum_{i=1}^{4}\beta iXi\;+\;\sum_{i=1}^{4}\beta ii{Xi}^{2}\;+\;\sum_{i=1}^{3}\times \sum_{j=1+1}^{4}\beta ijXiJi,$$

where Y represents the predicted flocculating activity, Xi and Ji indicated the independent parameters (pH, shaking time, concentration, and cations), and $\beta_{0}$, βi, βii, and βij are model constant, linear, squared, and 2-way interaction effects, respectively [25]. The ranges for the independent parameters [CaCl_2_ concentration (1.0–3.0% *w*/*v*), bioflocculant dosage (0.2–1.0 mg/mL), pH (4–10), and agitation time (1–10 min)] were selected based on preliminary screening experiments and prior reports on microbial bioflocculants.

### 2.3. Application of the Bioflocculants in Wastewater Treatment

Both the purified bacterial and fungal bioflocculants were used to treat wastewater from a local wastewater treatment plant using the optimum operational condition identified by RSM. The pH of the wastewater was adjusted to the optimum pH using 0.1 M HCl and 0.1 M NaOH. Chemical flocculants such as aluminium sulfate (alum) and polyaluminium chloride (PAC) were used as control treatments under the same optimum operational conditions (CaCl_2_ concentration, pH, dosage, and agitation time) determined for the bioflocculants by RSM. The removal efficiencies of the bioflocculants and chemical flocculants on chemical oxygen demand (COD) and turbidity were measured by the Spectroquant COD test kit (Merk KgaA, Darmstadt, Germany) and a Hanna HI801-01 turbidimeter (Hanna Instruments, Woonsocket, RI, USA), following the manufacturers’ instructions. The removal efficiency (RE) was calculated and expressed as percentage RE (%RE) using the formula:

%RE = (C_o_ − C)/C_o_ × 100, (4)

whereby C_o_ and C represented the values obtained before and after treatment, respectively [26].

### 2.4. Flocculation Mechanism of the Bioflocculants

Zeta potentials of different samples were measured to predict the flocculation mechanism of the bacterial and fungal bioflocculants using Zetasizer Nano (Malvern, UK). The samples included bioflocculant solution, bioflocculant solution combined with the cation, wastewater and flocs. The pH of the samples was adjusted to optimum operational pH using HCl or NaOH. A total volume of 2.5 mL was injected carefully into clear disposable cuvettes and read for zeta potentials at 25 °C with a count rate of 7.5 kcps. Three zeta runs were performed for each sample [27].

### 2.5. Data Analysis

All experiments were conducted in triplicate, and the results were presented as mean values with their corresponding standard deviations. Statistical analysis was performed using one-way analysis of variance (ANOVA) with GraphPad Prism™ 9, considering a *p*-value less than 0.05 as the threshold for statistical significance. Tukey’s Honest Significant Difference test was applied to compare group means for significant differences. Variations between the predicted and actual flocculating activity were evaluated using an independent Student’s *t*-test, performed at a 95% confidence level [28]. RSM data was analysed using Minitab software (trial version 21.2), with ANOVA employed to examine the linear, quadratic, and interaction effects of selected factors on the response variable. Model validity and significance were assessed through statistical metrics, including the coefficient of determination (R^2^), adjusted R^2^, and lack-of-fit tests. The response variable’s significance was confirmed at a 95% confidence level, ensuring *p* values remained below 0.05.

## 3. Results and Discussion

### 3.1. Characterization of the Extracted Bioflocculants

#### 3.1.1. Morphology of Bioflocculants Kp1 and Mg1

**Figure 1:**
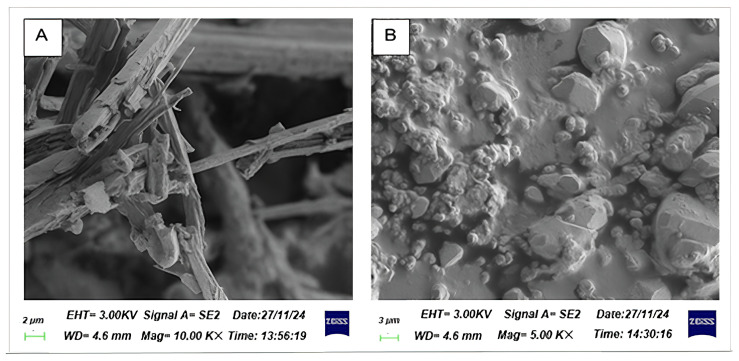
Surface morphology of bioflocculant Kp1 (**A**) and Mg1 (**B**).

The surface morphology of bioflocculants Kp1 and Mg1 was evaluated and Kp1 appeared white (Kp1) in colour, whereas Mg1 was a light brown powder. Moreover, bioflocculant Kp1 displayed a fibrous and irregular structure across all magnifications, suggesting a predominantly amorphous architecture consistent with polymeric or organic bioflocculants (Figure 1A). This fibrous morphology is indicative of its high polysaccharide content and reflects its potential to form an extended network conducive to effective particle entrapment in flocculation processes [29]. Mg1 illustrated a distinctly granular and crystalline surface morphology, suggesting the presence of structured mineral phases, corroborating its lower protein and polysaccharide content but a higher fraction of diverse biomolecules, potentially inclusive of mineral-associated components (Figure 1B) [30].

#### 3.1.2. Elemental Composition of Kp1 and Mg1

**Figure 2:**
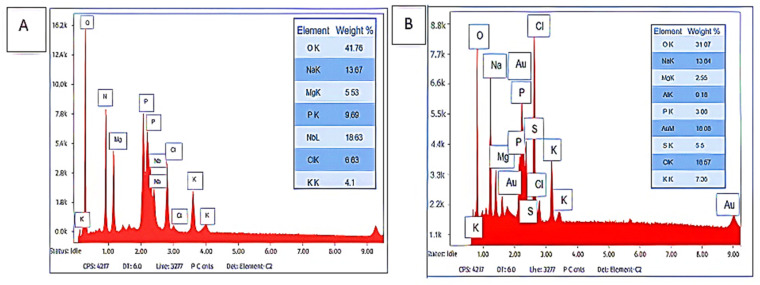
Elemental composition of Kp1 (**A**) and Mg1 (**B**).

Figure 2 demonstrates the detailed elemental mass ratios of the purified bioflocculants Kp1 and Mg1. The most predominant trace element in bioflocculant Kp1 was niobium (Nb) with 21%, followed by phosphorus (P) and oxygen (O_2_) with 20% and 16%, respectively. Potassium (K) and magnesium (Mg) were the least abundant at 10% (Figure 2A). In contrast, bioflocculant Mg1 was dominated by chlorine (Cl), accounting for 17%, whereas Mg and sodium (Na) exhibited the lowest proportions, with 5% and 8%, respectively (Figure 2B). The dominance of Nb and P as the primary components in Kp1 suggests that this bioflocculant contains a significant proportion of elements commonly associated with enhanced flocculating abilities, owing to their chemical reactivity and capacity to form stable complexes [31]. The presence of Nb in Kp1 aligns with the findings of Li et al. [29] who reported that Nb-enriched bioflocculants exhibit superior performance in heavy metal removal from wastewater. However, the relatively high Nb content detected in Kp1 may partly reflect potential contamination or signal interference. Possible sources include residual metallic particles within the SEM chamber, scattering from the gold coating, or elemental contributions from the aluminum stub used for mounting. The abundance of P may contribute to strengthening the polymeric network in Kp1, thereby promoting efficient particle aggregation. Conversely, the elemental profile of Mg1, characterized by the dominance of Cl^−^ and the lower proportions of Mg and Na, indicates a distinct compositional pattern. The presence of sulphur within Mg1 further suggests its potential suitability for specialised flocculation processes, such as metal recovery or bioremediation, consistent with the observations of Wang et al. [32]. The relatively high oxygen content in both bioflocculants underscores the role of the hydroxyl group in bioflocculation, as these are commonly involved in hydrogen bonding and particle capture.

#### 3.1.3. Functional Groups of Bioflocculants Kp1 and Mg1

**Figure 3:**
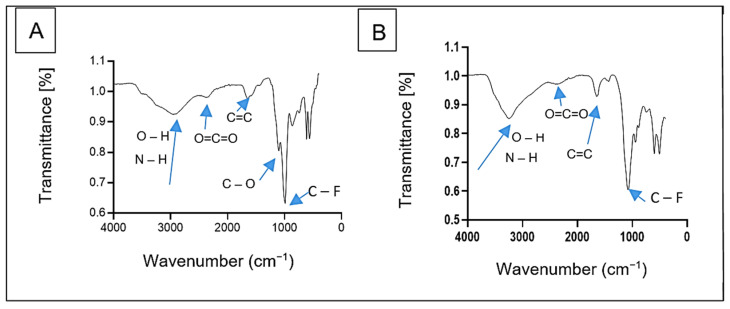
FTIR spectra of bioflocculant Kp1 (**A**) and Mg1 (**B**).

The FTIR spectra of bioflocculants Kp1 and Mg1 revealed distinct functional groups critical to their chemical structure and functionality (Figure 3). The spectra were recorded using a fixed resolution of 4 cm^−1^, ensuring consistent and reliable peak identification across samples. Bioflocculant Kp1 displayed characteristic peaks at 2944 cm^−1^, 2371 cm^−1^, 1657 cm^−1^, 1120 cm^−1^, and 1000 cm^−1^ (Figure 3A). These peaks were, respectively, attributed to overlapping O–H and N–H stretching vibrations, O=C=O stretching, C=C stretching, as well as C–O and C–F stretching vibrations. Similarly, bioflocculant Mg1 exhibited absorption bands at 3260 cm^−1^, 2388 cm^−1^, 1648 cm^−1^, and 1093 cm^−1^ (Figure 3B). These correspond to overlapping O–H and N–H stretching vibrations, O=C=O stretching, C=C stretching, and C–O stretching vibrations.

The hydroxyl and amine groups found in both Kp1 and Mg1 provide binding sites for ion bridging, a critical mechanism for bioflocculation [33]. Moreover, the presence of alkene and alkyl fluoride groups further confirmed that the significant components of the bioflocculants were polysaccharides and proteins [34]. The observations were consistent with the findings by Agunbiade et al. [35], who identified hydroxyl and amine groups as key functional groups in bioflocculants.

#### 3.1.4. Crystallinity of Bioflocculants Kp1 and Mg1

**Figure 4:**
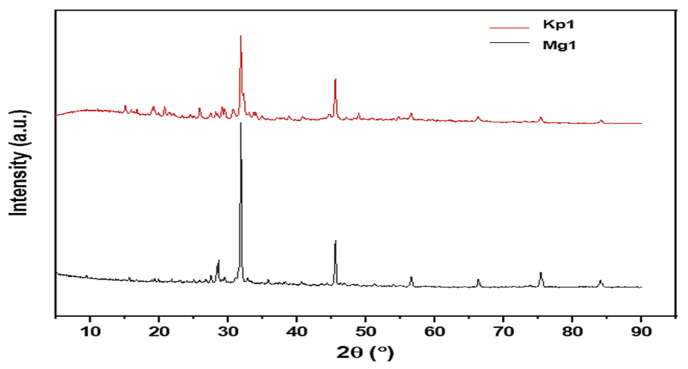
Crystallinity profiles of bioflocculants Kp1 and Mg1.

X-ray diffraction (XRD) analysis was conducted to elucidate the crystalline and structural characteristics of the bioflocculants Kp1 and Mg1 (Figure 4). The XRD pattern of Kp1 exhibited two prominent diffraction peaks at 2θ = 33° and 46° (Figure 4), which can be accurately indexed to the (104) and (200) crystal planes of calcium carbonate (CaCO_3_) in its calcite phase (JCPDS 05-0586). These sharp reflections confirm the presence of crystalline mineral residues, likely incorporated during bioflocculant biosynthesis or through microbial mineralization processes. In addition, broad diffraction features observed at 2θ = 14°, 18°, 26°, 48°, 58°, 68°, 77°, and 85° indicated the presence of amorphous organic macromolecular components, such as polysaccharides and proteins, which are typical of biopolymeric matrices lacking long-range order. The XRD pattern of Mg1 displayed distinct sharp peaks at 2θ = 28°, 32°, and 46° (Figure 4), corresponding to the (111), (200), and (220) planes of magnesium oxide (MgO) (JCPDS 45-0946), confirming the formation of a well-defined crystalline inorganic phase. Additional broad halos at 2θ = 57°, 67°, 76°, and 84° further suggest the coexistence of amorphous organic regions intertwined with these crystalline domains. Collectively, the XRD profiles of Kp1 and Mg1 demonstrated their semi-crystalline nature, comprising both ordered crystalline structures and disordered amorphous phases. The coexistence of these two structural domains is functionally advantageous: the crystalline regions impart mechanical strength and structural rigidity, while the amorphous regions contribute flexibility to the biopolymer matrix. Although no direct mechanical testing was performed in this study, the diffraction data implies that this semi-crystalline configuration could enhance the bioflocculants’ stability and durability. This observation aligned with the findings of Maćczak et al. [36], who reported that bioflocculants with mixed crystalline–amorphous architectures exhibit improved mechanical integrity due to synergistic reinforcement between their ordered and disordered molecular phases.

#### 3.1.5. Pyrolysis Profiles of Bioflocculants Kp1 and Mg1

**Figure 5:**
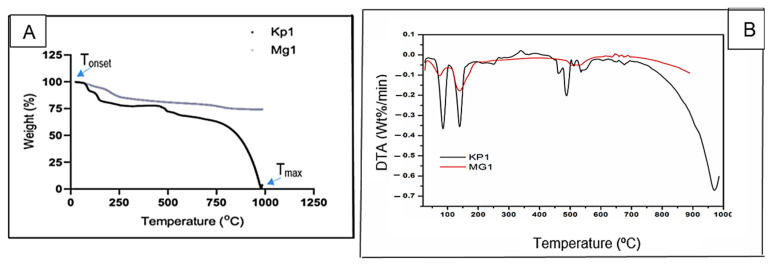
TGA (**A**) and DTA (**B**) spectra of bioflocculants Kp1 and Mg1.

The thermal degradation profiles of bioflocculants Kp1 and Mg1 as a function of temperature are illustrated in Figure 5. The bioflocculant Kp1 exhibited an initial weight loss of approximately 22% within the temperature range of 0–130 °C, followed by a gradual decomposition phase resulting in an additional 8% weight loss between 130 and 500 °C. By 1000 °C, Kp1 had undergone complete thermal degradation, leaving no residual weight. In contrast, Mg1 displayed a 20% weight loss from 0 to 260 °C, followed by a minor 3% loss over the extended range of 260–1000 °C, ultimately retaining 77% of its original weight. The initial weight loss observed in both was likely due to the evaporation of moisture and volatile organic compounds [37], while the subsequent slower degradation phase corresponded to the breakdown of low-molecular-weight organic constituents. The complete degradation of Kp1 by 1000 °C indicated the absence of thermally stable inorganic components [24], suggesting a predominantly organic composition. Conversely, the minor additional weight loss and high residual mass of Mg1 suggest the presence of thermally stable inorganic or carbonaceous compounds [35]. The relatively high thermal stability of Mg1 compared to Kp1 implies that Mg1 may be more suitable for high-temperature applications such as wastewater treatment processes, where thermal resilience enhances operational stability. These observations, however, contrast with previous findings on *Klebsiella*-derived bioflocculants such as *K. pneumoniae* YZ-6, which demonstrated higher thermal stability [38], as well as reports on several fungal bioflocculants that exhibited enhanced resistance to thermal degradation [3,39].

#### 3.1.6. Biosafety Assessment of the Purified Bioflocculants

**Figure 6:**
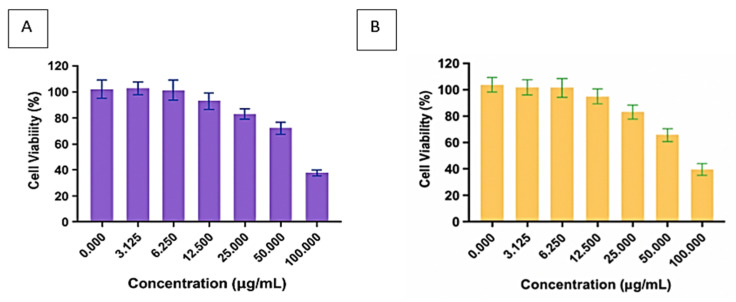
Cytotoxic effect of bioflocculants Kp1 (**A**) and Mg1 (**B**) on CACO-2 cells.

Figure 6 illustrates the cytotoxic effects of the bioflocculants Kp1 and Mg1 on CACO-2 cell lines, revealing a clear concentration-dependent relationship. As the concentrations of both bioflocculants increased, cell viability significantly decreased, indicating potential cytotoxicity at higher doses. Specifically, cell viability for Kp1 declined from 93.18% at 3.13 µg/mL to 39.36% at 100 µg/mL, while Mg1 showed a similar trend, with viability dropping from 98.25 to 38.97% across the same concentration range. These results imply that low concentrations (≤3.13 µg/mL) of Kp1 and Mg1 exhibit minimal cytotoxicity, maintaining over 90% cell viability. This is particularly relevant for wastewater treatment applications, where bioflocculants are typically effective at low dosages to achieve high flocculation efficiency [40]. Thus, at the operational concentrations commonly employed in wastewater treatment, Kp1 and Mg1 are likely to be biocompatible and environmentally safe, minimising ecological and human health risks. However, caution should be exercised at elevated concentrations, especially during large-scale production, handling, or accidental spillage, as cytotoxic effects may become significant.

### 3.2. Optimisation of Cations

**Figure 7:**
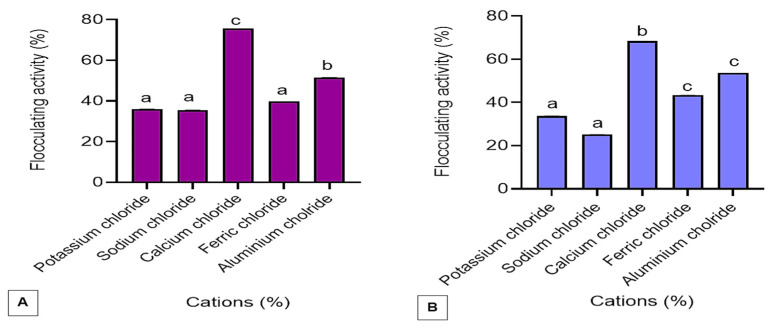
The effect of various cations on the %FA of the bioflocculant Kp1 (**A**) and Mg1 (**B**). The alphabets signify statistical significance at *p* < 0.05.

The effect of various cations on the flocculating activity of the bioflocculants Kp1 and Mg1 is presented in Figure 7. The monovalent cations (KCl and NaCl) yielded the lowest flocculating activities of 35.99% and 35.53% for Kp1 and Mg1, respectively. In contrast, trivalent cations (AlCl_3_ and FeCl_3_) produced moderate flocculating activities, while the divalent cation (CaCl_2_) significantly (*p* < 0.05) enhanced flocculating performance, achieving 75.60% for Kp1 and 68.38% for Mg1. Consequently, CaCl_2_ was selected for subsequent assays and concentration optimisation. The observed variation in flocculating activity highlights the strong dependence of bioflocculant performance on the type and valence of metal cations present. The relatively low activities in the presence of monovalent cations may be attributed to their lower charge density and weaker electrostatic bridging capacity between suspended particles and the functional groups of the bioflocculants [41]. In contrast, the addition of divalent cations such as Ca^2+^ markedly improved flocculating activity, likely due to their higher charge density and stronger ionic bridging potential, which facilitate aggregation and stability of flocs [30].

### 3.3. RSM Optimisation of Operational Conditions

#### 3.3.1. RSM Optimisation of Operational Conditions of Kp1

The bioflocculant Kp1 demonstrated an outstanding flocculating activity of 90.11% under the following experimental conditions: a CaCl_2_ concentration of 2% (*w*/*v*), high dosage of 1.0 mg/mL, alkaline pH of 10 and agitation time of 5 min (Run 4, Table 1). Conversely, the lowest flocculating activity of 70.33% was observed when a CaCl_2_ concentration of 2% (*w*/*v*), low dosage of 0.2 mg/mL, acidic pH of 4 relatively and medium agitation time of 5 min were used (Run 1, Table 1). These results highlighted the importance of optimal bioflocculant dosage and CaCl_2_ concentration, which enhanced the stability of the flocs. Furthermore, the preferred pH levels suggested that this bioflocculant may be better suited for environments with an alkaline pH, where its flocculating activity is enhanced, likely due to better electrostatic interactions between the bioflocculant and suspended particles [33]. Kp1 exhibited the lowest flocculating activity under moderate CaCl_2_ concentration, low flocculant dosage, acidic pH, and moderate agitation. The decreased flocculation observed under these conditions was likely due to the low dosage of bioflocculant added, which provided fewer binding sites for pollutants. As a result, insufficient particle aggregation occurred, leading to poor floc formation [40]. Moreover, the acidic pH likely altered the ionisation state of Kp1 molecules, diminishing their capacity to neutralise the surface charges of suspended particles, thereby impairing their aggregation efficiency [5].

#### 3.3.2. RSM Optimisation of Operational Conditions of Mg1

The bioflocculant Mg1 produced a maximum flocculating activity of 92.65% under the optimised conditions comprising a 2% (*w*/*v*) concentration, moderate levels of CaCl_2_, a high dosage of 1.0 mg/mL, an acidic pH of 4, and an agitation time of 5 min (Run 2, Table 2). The acidic environment may have enhanced electrostatic interactions, promoting floc aggregation [42], whereas high dosage and medium CaCl_2_ concentration likely improved the bridging and charge neutralisation processes, resulting in high flocculating activity [43]. In contrast, the lowest flocculating activity of 72.55% was recorded in experimental run 22, with a CaCl_2_ concentration of 2% (*w*/*v*), dosage of 0.6 mg/mL, alkaline pH of 10 and low agitation time of 3 min. The reduced flocculation under medium CaCl_2_ concentration, medium dosage, alkaline pH and low agitation time was likely due to the impediment of the flocculant’s ability to interact effectively with suspended particles. Additionally, the reduced agitation time could have prevented proper mixing, limiting the bioflocculant’s contact with the particles and decreasing its overall effectiveness.

### 3.4. RSM Contour and Surface Plots for Bioflocculant Kp1

#### 3.4.1. Interaction Effect of pH and Dosage on Flocculating Activity

**Figure 8:**
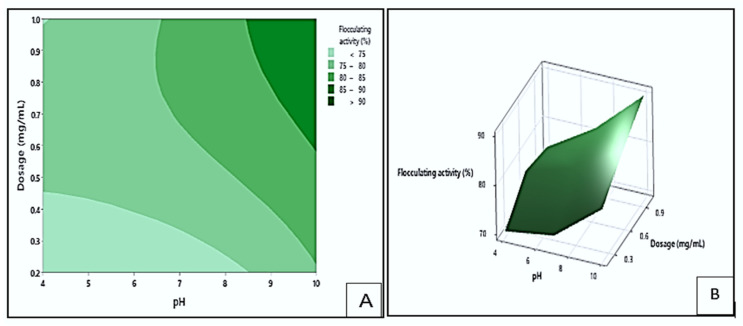
The contour (**A**) and surface plots (**B**), show the interaction between dosage and pH and its effect on flocculating activity by bioflocculant Kp1.

Figure 8 presents the contour (A) and response surface (B) plots illustrating the impact of dosage and pH on the flocculating activity of the bioflocculant Kp1. The darker green area in the contour plot indicates the region with the highest possible flocculating activity values, exceeding 90%. This maximum activity could be achieved within a dosage range of 0.6 to 1.0 mg/mL and a pH range of 8.6 to 10. In contrast, the light green region represents the conditions with the lowest flocculation (<75%), which could occur within dosage sizes of 0.2 to 0.45 mg/mL and pH ranges of 4 to 8.5. These observations suggested that alkaline pH and higher dosage levels were favourable for effective aggregation of particles when using bioflocculant Kp1. The positive influence of increased dosage is consistent with the study by Hadiyanto et al. [44], which indicated that an optimal bioflocculant concentration enhances particle aggregation by increasing the availability of active functional groups that interact with suspended particles.

#### 3.4.2. Interaction Effect of Dosage and CaCl_2_ on Flocculating Activity

**Figure 9:**
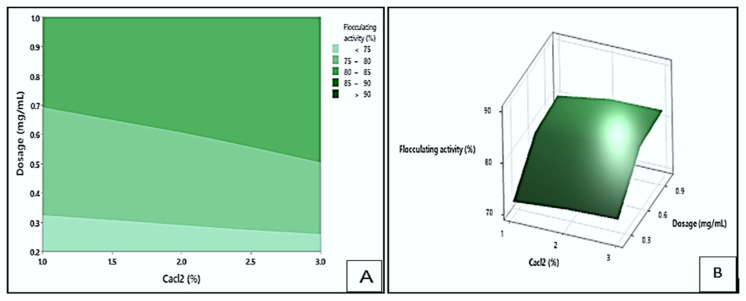
The contour (**A**) and response surface (**B**) plots display the interaction between dosage and CaCl_2_ concentration and its effect on flocculating activity by bioflocculant Kp1.

Figure 9 shows the contour (A) and response surface (B) plots depicting the effect of dosage and CaCl_2_ concentration on the flocculating activity of the bioflocculant Kp1. The plots highlighted that the highest flocculating activity values (greater than 90%) could be obtained at the darkest green area. These optimal values could be achieved within a dosage range of 0.69 to 1.0 mg/mL and a CaCl_2_ concentration between 1.0 and 3.0% (*w*/*v*). The lowest flocculating activity (below 75%) is depicted by the lightest green area and could be obtained when the dosage ranged between 0.2 and 0.32 mg/mL, and the CaCl_2_ concentration maintained between 1.0 and 3.0% (*w*/*v*). These results implied that both dosage and CaCl_2_ concentration significantly influenced the flocculating activity of Kp1. The optimal combination of these parameters likely enhanced charge neutralisation and bridging between particles, promoting effective floc formation [45].

#### 3.4.3. Interaction Effect of pH and CaCl_2_ on Flocculating Activity

**Figure 10:**
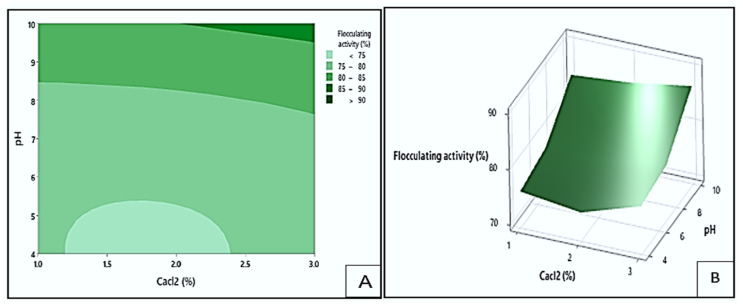
The contour (**A**) and surface plots (**B**), depict Kp1′s flocculating activity as a function of the interaction between pH and CaCl_2_ concentration.

Figure 10 displays contour (A) and response surface (B) illustrating the impact of pH and CaCl_2_ concentration on the flocculating activity of Kp1. In the contour plot, the dark green area represented the area where the highest flocculating activity (>90%) could be obtained at pH levels between 9.5 and 10 and CaCl_2_ concentrations ranging from 2.1 to 3.0% (*w*/*v*). The light green region indicated the region with the lowest flocculation values (<75%). The lowest flocculation could be obtained at pH levels between 4.0 and 5.4 and CaCl_2_ concentrations between 1.2 and 2.4% (*w*/*v*). This suggested that the interplay between alkaline pH and high CaCl_2_ concentration positively contributed to flocculation efficiency. Under slightly alkaline conditions, Kp1 likely maintained optimal charge characteristics, promoting stronger interactions with colloids and improving floc formation. This finding aligns with the work of Srinivasan et al. [46], who observed that the bioflocculant from *K. pneumoniae* exhibited high performance within a similar pH range. The calcium ions likely interacted with both the bioflocculant Kp1 and colloidal particles at optimal concentrations, stabilising flocs and facilitating particle aggregation. This observation agrees with the findings of previous studies [47], which demonstrated that calcium ions promote charge neutralisation and bridging between bioflocculant polymers and suspended solids, thereby enhancing flocculation efficiency.

### 3.5. RSM Contour and Surface Plots for Bioflocculant Mg1

#### 3.5.1. Interaction Effect of Dosage and pH on Flocculating Activity

**Figure 11:**
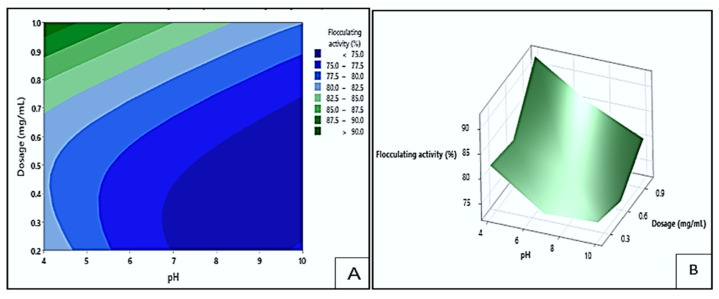
The contour (**A**) and surface plots (**B**) showing Mg1′s flocculating activity as a result of the interaction between dosage size and pH.

The effect of dosage and pH on flocculating activity by bioflocculant Mg1 is displayed in Figure 11. The darkest green area of the contour plot (A) indicated the area where the highest flocculating activity values exceeding 90% could be obtained. This could occur at the pH levels of 4.0 to 4.5 and dosage sizes between 0.95 and 1.0 mg/mL. The darkest blue area represented the lowest flocculation (<75%), which could be found at pH levels of 7 to 10 and dosage sizes ranging from 0.2 to 0.68 mg/mL. These findings showed that higher dosage ranges and acidic pH levels increased flocculating efficiency by maintaining optimal charge characteristics and increasing the number of active binding sites for particle interaction [48]. Agunbiade et al. [33] similarly confirmed that higher bioflocculant dosages enhance flocculation by promoting particle bridging and aggregation.

#### 3.5.2. Interaction Effect of Dosage and CaCl_2_ on Flocculating Activity

**Figure 12:**
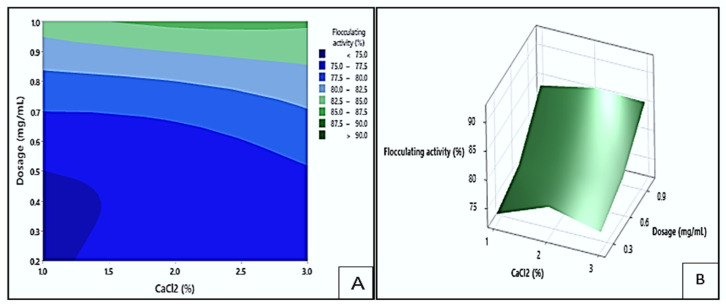
The contour plot (**A**) and surface plots (**B**) illustrating the flocculating activity of Mg1 as a result of the interaction between dosage size and CaCl_2_ concentration.

Figure 12 presents contour (A) and response surface (B) plots showing the effect of dosage and CaCl_2_ concentration on the flocculation. In the contour plot, the dark green area highlighted the highest flocculating activity, exceeding 90%. These values could be achieved with dosage levels between 0.98 and 1.0 mg/mL and CaCl_2_ concentrations ranging from 1.6 to 3.0% (*w*/*v*). On the other hand, the dark blue region represented the lowest response values (<75%), which occurred at dosage levels between 0.2 and 0.5 mg/mL and CaCl_2_ concentrations between 1.0 and 1.4% (*w*/*v*). These findings implied that the flocculating efficiency of Mg1 was markedly affected by both dosage and CaCl_2_ concentration. Enhanced flocculating efficiency was observed under higher dosage and CaCl_2_ levels, whereas lower parameter values corresponded to reduced activity. This trend aligns with previous reports highlighting that appropriate bioflocculant dosage and divalent cation concentration facilitate optimal charge neutralisation and particle aggregation [45].

#### 3.5.3. Interaction Effect of pH and CaCl_2_ on Flocculating Activity

**Figure 13:**
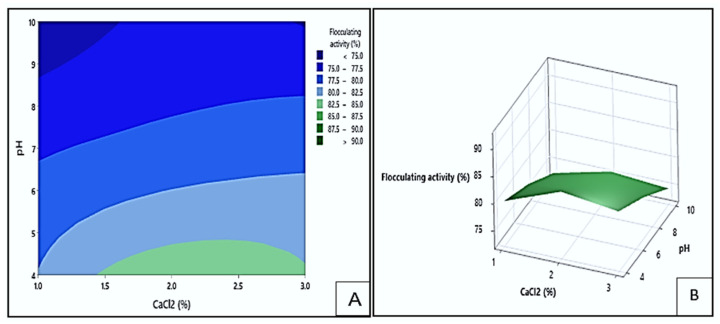
The contour (**A**) and surface plots (**B**) demonstrating the flocculating activity of Mg1 as influenced by the interaction between pH and CaCl_2_.

The impact of pH and CaCl_2_ concentration on the flocculating activity of the bioflocculant Mg1 is shown in Figure 13 by the contour (A) and response surface (B) plots. The green site of the contour plot indicated the area where the highest flocculating activities surpassing 82.5% could be obtained. These values could be observed at pH levels between 4.0 and 4.7 and CaCl_2_ concentration range of 1.4 to 3.0% (*w*/*v*). On the other hand, the darkest blue areas represented the lowest flocculating activities below 75%, which occurred at pH levels between 8.7 and 10 and CaCl_2_ concentration ranging from 1.0 to 1.6% (*w*/*v*). Optimal pH and CaCl_2_ concentrations likely enhanced particle aggregation by neutralising repulsive forces between negatively charged particles and bioflocculant molecules. However, deviations from these optimal ranges hindered flocculation efficiency. This observation is consistent with the report by Mohammed et al. [49], who found that calcium ion concentration significantly influences flocculation performance by affecting the stability and compactness of formed flocs.

### 3.6. Response Optimiser

**Figure 14:**
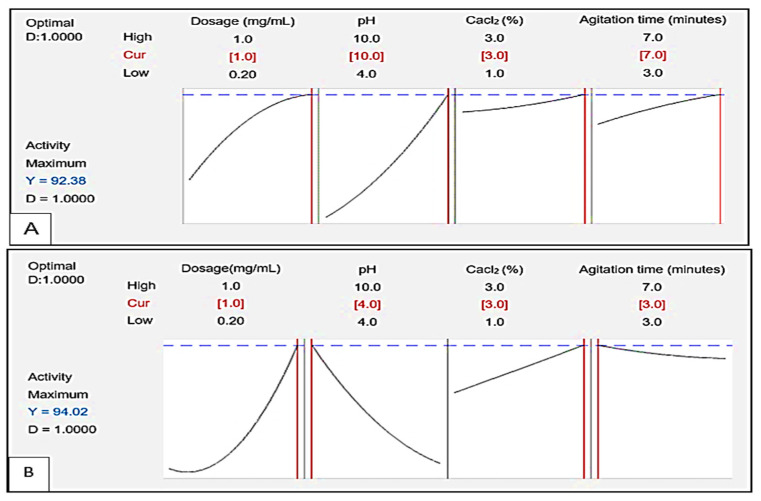
Process optimisation graphs for a target value (flocculating activity) to be achieved by Kp1 (**A**) and Mg1 (**B**) with dosage, pH, CaCl_2_ concentration and agitation time as significant factors.

Figure 14 displays the response optimiser showing the desirability (D) and the optimal conditions for flocculating activity of bioflocculants Kp1 and Mg1. The D function yielded a value of 1.00 for both Kp1 (Figure 14A) and Mg1 (Figure 14B). The predicted optimal conditions for maximum flocculating activity are highlighted in red and indicated the highest efficiencies of 92.38 and 94.02% for Kp1 and Mg1, respectively. The ideal conditions for Kp1 included a high dosage of 1.0 mg/mL, alkaline pH of 10, high CaCl_2_ concentration of 3.0% (*w*/*v*) and maximum agitation time of 7 min. For Mg1, the optimal conditions included a high dosage of 1.0 mg/mL, acidic pH of 4, high CaCl_2_ concentration of 3.0% (*w*/*v*), and minimal agitation time of 3 min.

### 3.7. Verification of the Predicted Efficiency by Response Optimiser

Laboratory validation of the predicted conditions by response optimiser showed insignificant differences in flocculating activities for both bioflocculant Kp1 and Mg1. Bioflocculant Kp1 revealed a maximum flocculating activity of 90.11% whereas bioflocculant Mg1 gave 92.65% flocculating activity. These results further supported the robustness and accuracy of the models. They were coincided with other findings, which showed insignificant differences between the predicted and the actual flocculating activity values, further highlighting the reliability of RSM [50,51].

### 3.8. Regression Equations of the Models

#### 3.8.1. Regression Equations of the Model of Kp1

The model statistics were done to determine the best model representing the flocculating activity of the purified bioflocculant Kp1. The second-order response model that fitted well after the regression analysis was expressed as:

Y = 75.09 + 21.84 X_1_ + 3.28 X_2_ + 0.88 X_3_ − 7.98 X_4_ + 16.09 X_1_^2^ + 0.2000 X_2_^2^ + 0.330 X_3_^2^ − 0.090 X_4_^2^ + 1.240 X_1_ × X_2_ + 0.39 X_1_ × X_3_ − 0.250X_1_ × X_4_ + 0.054 X_2_ × X_3_ − 0.2617 X_2_ × X_4_ − 0.026 X_3_ × X_4_, (5)

where Y denotes the %FA and X_1_, X_2_, X_3_ and X_4_ represent the dosage (mg/L), pH, CaCl_2_% (*w*/*v*) and agitation time (minutes), respectively.

The positive coefficients for dosage (X_1_), pH (X_2_), and CaCl_2_ concentration (X_3_) indicated that an increase in these parameters enhanced the flocculating activity of Kp1. This enhancement suggested that higher bioflocculant dosages and favourable ionic conditions could improve particle aggregation, resulting in more efficient floc formation and sedimentation. These findings were inconsistent with previous research on microbial flocculants, which highlighted that optimal dosages result in more efficient flocculation. Furthermore, a study by Ding et al. [42] supported the notion that optimising pH levels and incorporating adequate CaCl_2_ concentrations can significantly improve flocculation. Conversely, the negative coefficient for agitation (X_4_) implied that excessive or prolonged agitation may have decreased flocculating efficiency [52], possibly because of the shear forces that destabilise the floc structure [53]. The positive interaction between dosage and CaCl_2_ concentration suggested that higher dosages and Ca^2+^ concentrations could work synergistically to improve flocculation. Additionally, the negative interaction between pH and agitation time (higher than the negative coefficient for agitation) implied that higher pH may alleviate the adverse effects of extended agitation.

#### 3.8.2. Regression Equations of the Model of Mg1

The model statistics were performed to identify the optimal model representing the flocculating activity of the purified bioflocculant Mg1. The second-order response model, which best fitted the data after regression analysis was expressed as:

Y = 85.93 + 12.34 X_1_ + 3.30 X_2_ + 3.74 X_3_ − 0.50 X_4_ + 26.17 X_1_^2^ + 0.1884 X_2_^2^ + 0.030 X_3_^2^ − 0.064 X_4_^2^ + 1.058 X_1_ × X_2_ + 0.14 X_1_ × X_3_ − 0.122 X_1_ × X_4_ + 0.078 X_2_ × X_3_ − 0.017 X_2_ × X_4_ − 0.469 X_3_ × X_4,_ (6)

where Y represents the flocculating activity (%) corresponding to the dosage (mg/L), pH, CaCl_2_% (*w*/*v*), and agitation time (minutes), respectively.

The positive coefficients for dosage (X_1_), pH (X_2_), and CaCl_2_ concentration (X_3_) suggested that increasing these variables likely enhanced flocculating activity, similar to the trend observed for Kp1. This finding aligns with prior research emphasising the role of optimal dosages, pH levels and cation concentration in flocculation efficiency. However, the negative coefficient for agitation (X_4_) indicated that increased agitation may have reduced flocculating activity likely due to the shear forces disrupting the floc structure [53]. The interaction between dosage and pH suggested that increasing dosage in slightly alkaline conditions could further enhance its flocculating activity. This phenomenon is supported by Ding et al. [42], who reported that pH adjustments can significantly impact flocculation. In contrast, the negative interaction between dosage and agitation time highlighted the need for careful control of both dosage and agitation time to prevent shear forces from disrupting the floc structures. This delicate balance is crucial for maximising flocculation performance and ensuring the stability of the flocs [53].

### 3.9. ANOVA Analysis of the Models

#### 3.9.1. ANOVA Analysis of the Model of Bioflocculant Kp1

The ANOVA analysis indicated that the quadratic model for bioflocculant Kp1 exhibited a high F-value of 35.68 and a significantly low *p*-value (*p* < 0.01, Table S2), confirming that the model effectively captured the variations in flocculating activity. The coefficient of determination (R^2^ = 0.9765) and adjusted R^2^ (0.9492) further demonstrated that the model explained a large proportion of the variability in the experimental data. Additionally, the lack-of-fit test yielded a *p*–value of 0.324, exceeding the 0.05 threshold, which confirmed that the model adequately fitted the data without significant deviation between predicted and observed values. These findings collectively validated the robustness and reliability of the quadratic model in describing the flocculating behaviour of Kp1 [47,49,50]. The linear terms [dosage (X_1_), pH (X_2_), and CaCl_2_ concentration (X_3_)] were statistically significant at the 5% level (*p* < 0.05), highlighting their crucial roles in determining the flocculating activity of Kp1. In contrast, agitation time (X_4_) was not significant (*p* = 0.060), suggesting that this variable exerted a minor effect on flocculation efficiency. The squared terms (X_1_^2^, X_2_^2^, X_3_^2^ and X_4_^2^) collectively produced an F–value of 15.60 with a *p*–value of 0.00, indicating significant non-linear relationship between the variables and the response (flocculating activity). Among these, dosage (X_1_^2^) and pH (X_2_^2^) were particularly significant (*p* < 0.05), implying non-linear effects of these variables on flocculating activity and emphasising the importance of optimising both dosage and pH for maximal performance. Two-way interaction effects were also observed, with X_1_ × X_2_ and X_2_ × X_4_ showing statistical significance (*p* < 0.05). These results indicated that the interaction between dosage and pH, as well as between pH and agitation time, significantly influenced flocculating activity. Such interactions suggest that pH mediates the synergistic relationship between flocculant dosage and mixing conditions, potentially affecting charge neutralisation and particle aggregation dynamics. Other interaction terms were not significant, implying that additional factor combinations did not substantially affect the flocculating performance of Kp1.

#### 3.9.2. ANOVA Analysis of the Model of Bioflocculant Mg1

The ANOVA and regression coefficients for the purified bioflocculant Mg1 are presented in Table S3. The quadratic model demonstrated a high F-value of 29.13 and a statistically significant *p*-value (*p* < 0.01), indicating that the model effectively described the relationship between the independent variables and flocculating activity. The coefficient of determination (R^2^ = 0.9714) and adjusted R^2^ (0.9381) values confirmed that the model explained a substantial proportion of the variability in the experimental data. Furthermore, the lack–of–fit test yielded a *p*-value of 0.374, which exceeded the 0.05 significance threshold, confirming that the model adequately fitted the data with no significant discrepancies between predicted and observed values. These findings collectively affirm the robustness and reliability of the quadratic model in predicting the flocculating behaviour of Mg1 [54,55]. The linear terms [dosage (X_1_), pH (X_2_), and CaCl_2_ concentration (X_3_)] were statistically significant at the 5% level (*p* < 0.05), emphasising their critical influence on flocculating activity. In contrast, agitation time (X_4_) was not significant (*p* = 0.183), suggesting a comparatively weaker effect on Mg1′s flocculation performance. The squared terms (X_1_^2^, X_2_^2^, X_3_^2^, and X_4_^2^) collectively yielded an F–value of 19.56 and a *p*-value of 0.00, confirming a significant non-linear relationship between the variables and the response. Among these, dosage (X_1_^2^) and pH (X_2_^2^) were particularly significant (*p* = 0.00), indicating that variations in these parameters had non-linear effects on flocculating activity, thus underscoring the importance of their optimisation for achieving maximum flocculation efficiency. Unlike the model for Kp1, the two-way interaction terms (X_1_ × X_2_, X_1_ × X_3_, X_1_ × X_4_, X_2_ × X_3_, X_2_ × X_4_ and X_3_ × X_4_) did not exhibit statistical significance (*p* > 0.05). This finding suggests that the combined effects of dosage, pH, CaCl_2_ concentration, and agitation time did not have a substantial influence on Mg1′s flocculating activity. Therefore, the primary determinants of Mg1′s performance were individual parameter effects, particularly dosage and pH, rather than synergistic interactions between variables.

### 3.10. Application of Purified Bioflocculants in Wastewater Treatment

**Figure 15:**
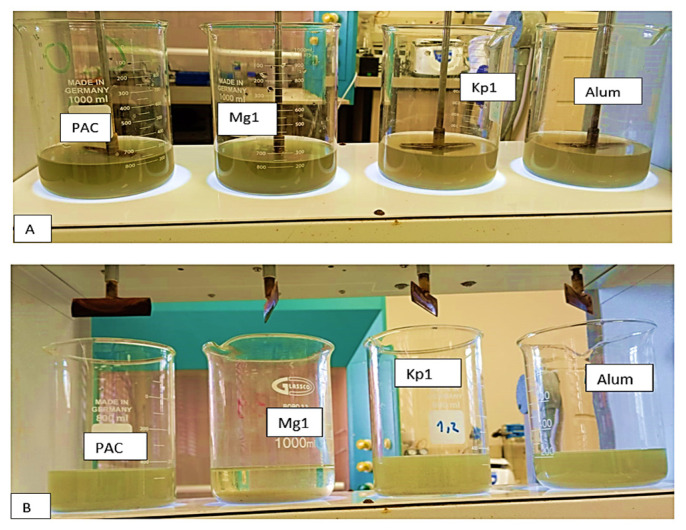
(**A**) The removal efficiencies of the bioflocculants Kp1 and Mg1 on COD and turbidity compared to PAC and aluminium sulfate under suboptimal conditions; (**B**) The removal efficiencies of the bioflocculants Kp1 and Mg1 on COD and turbidity compared to PAC and aluminium sulfate under optimal conditions.

The removal efficiencies of the bioflocculants Kp1 and Mg1 on COD and turbidity were compared to PAC and aluminium sulfate, as presented in Table 3. The COD removal efficiency of the bioflocculant Kp1 was comparably similar to those of other flocculants (*p* value > 0.05). However, the efficiency of bioflocculant Mg1 was significantly higher (93.12%) than those of the chemical flocculants (Figure 15). Furthermore, both bioflocculants Kp1 and Mg1 demonstrated notably higher removal efficiencies on turbidity compared to the chemical flocculants. This observation supported the idea that bioflocculants have the potential to be used as effective alternatives to chemical flocculants. These findings aligned with those observed by Mburu et al. [56] and De La Cruz-Noriega et al. [57], where microbial flocculants achieved significantly high removal efficiencies on pollutants in wastewater.

### 3.11. Evaluation of Flocculation Mechanisms of Bioflocculants Kp1 and Mg1

Table 4 presents the zeta potentials of various samples, including the bioflocculant solutions (Kp1 and Mg1), their respective mixtures with CaCl_2_, raw wastewater, and the resulting flocs. The addition of Ca^2+^ to the bioflocculant solutions led to a notable increase in zeta potential values. Specifically, the zeta potential of the Kp1 solution increased from 11.90 ± 0.87 to 22.93 ± 1.19 mV, while the Mg1 solution exhibited a more substantial rise from −0.3 ± 2.44 mV to 16.9 ± 0.6 mV. The zeta potential for Kp1 suggested that this bioflocculant operated primarily through charge neutralisation. In this process, the positively charged bioflocculant was likely electrostatically adsorbed onto the negatively charged colloidal wastewater particles. This neutralisation reduced repulsive forces and promoted aggregation. Moreover, the high zeta potential of the bioflocculant solution plus CaCl_2_ in comparison to the bioflocculant solution, implied that CaCl_2_ likely destabilised the solution by reducing the electrical double layer thickness, thus facilitating stronger adhesion of Kp1 to the colloids in solution [47]. Interestingly, the findings in this study contradicted most observations in literature, whereby microbial bioflocculants mainly utilised the bridging mechanism to reduce pollutants in solutions [27,58]. The differences might be owed to the chemical composition of the bioflocculants, which significantly affects their overall net charge, consequently influencing their flocculation mechanisms. Bioflocculant Mg1 displayed effectiveness through inter-particle bridging, where the negatively charged bioflocculant was most probably adsorbed onto the surface of multiple colloidal wastewater particles, effectively linking particles together. The high zeta potential value of the bioflocculant solution plus CaCl_2_ in comparison to the bioflocculant solution indicated that Ca^2+^ served as a crosslinking agent, reducing the negative charge of both the bioflocculant and colloidal wastewater pollutants, consequently enhancing large floc formation [59]. The findings aligned with most studies, which highlighted that most microbial flocculants exert flocculation through bridging mediated by cations [4,60].

## 4. Conclusions

The comprehensive characterisation of the bioflocculants Kp1 and Mg1 highlighted significant structural and functional variations that influence their flocculation behavior. SEM observations revealed that Kp1 exhibited a fibrous morphology, whereas Mg1 displayed a granular texture, indicative of differences in their aggregation dynamics. FTIR analysis confirmed the presence of hydroxyl, amine, and alkene functional groups, which serve as active sites facilitating particle adsorption and floc formation. The XRD patterns demonstrated that both bioflocculants possess a semi-crystalline nature, characterised by a coexistence of amorphous and crystalline domains. Furthermore, TGA analysis showed that Kp1 exhibited lower thermal stability compared to Mg1, which retained greater thermal resistance under elevated temperatures. Optimisation of operational parameters significantly improved the removal efficiencies of chemical oxygen demand (COD) and turbidity for both bioflocculants. Mechanistic analysis suggested that Kp1 induces flocculation primarily through a charge neutralisation mechanism, promoting the aggregation of oppositely charged colloidal particles, while Mg1 operates through a polymer bridging mechanism, establishing inter-particle linkages that enhance floc strength and sedimentation efficiency. Overall, the inter-relationship between the structural and functional properties of polymeric bioflocculants and their potential application in sustainable wastewater treatment was highlighted in this study. The cytotoxic effect of these bioflocculants at increased concentrations warrants cautious handling and application in lower doses. Moreover, future studies should focus on scaling up production processes, elucidating molecular-level interactions, and evaluating performance under real environmental and industrial conditions to enable practical implementation.

## Figures and Tables

**Table 1:** Optimisation of operational condition of bioflocculant Kp1.

Experimental Run	Independent Variables				Flocculating Activity (%)	
	CaCl_2_% (*w*/*v*)	Dosage (mg/mL)	pH	Agitation Time (Minutes)	Predicted FA ± SD	Actual FA ± SD
1	2	0.2	4	5	70.55 ± 0.04	70.33 ± 0.06
2	2	1.0	4	5	75.72 ± 0.58	74.43 ± 0.01
3	2	0.2	10	5	77.84 ± 0.32	79.45 ± 0.04
4	2	1.0	10	5	89.52 ± 0.48	90.11 ± 0.01
5	1	0.6	7	3	77.62 ± 0.23	78.14 ± 0.01
6	3	0.6	7	3	79.47 ± 0.09	79.73 ± 0.01
7	1	0.6	7	7	78.95 ± 0.05	79.14 ± 0.02
8	3	0.6	7	7	80.53 ± 0.17	80.06 ± 0.02
9	2	0.2	7	3	70.85 ± 0.26	70.60 ± 0.01
10	2	1.0	7	3	80.00 ± 0.04	80.73 ± 0.02
11	2	0.2	7	7	72.43 ± 0.50	71.10 ± 0.03
12	2	1.0	7	7	81.20 ± 0.32	81.57 ± 0.02
13	1	0.6	4	5	75.40 ± 0.11	75.04 ± 0.01
14	1	0.6	10	5	85.12 ± 0.14	84.92 ± 0.01
15	3	0.6	4	5	77.03 ± 0.06	77.63 ± 0.02
16	3	0.6	10	5	87.18 ± 0.30	86.51 ± 0.01
17	1	0.6	7	5	71.90 ± 0.08	72.02 ± 0.01
18	1	0.2	7	5	80.17 ± 0.07	80.15 ± 0.00
19	3	1.0	7	5	73.32 ± 0.07	73.11 ± 0.01
20	3	0.2	7	5	82.17 ± 0.13	81.85 ± 0.00
21	2	1.0	4	3	76.48 ± 0.06	76.71 ± 0.00
22	2	0.6	10	3	83.27 ± 0.13	82.66 ± 0.00
23	2	0.6	4	7	74.67 ± 0.01	75.79 ± 0.00
24	2	0.6	10	7	87.89 ± 0.04	87.60 ± 0.00
25	2	0.6	7	5	79.25 ± 0.15	78.55 ± 0.00
26	2	0.6	7	5	79.27 ± 0.19	79.56 ± 0.00
27	2	0.6	7	5	79.45 ± 0.45	79.89 ± 0.00

**Table 2:** Optimisation of operational conditions of bioflocculant Mg1.

Experimental Run	Independent Variables				Flocculating Activity (%)	
	CaCl_2_ % (*w*/*v*)	Dosage (mg/mL)	pH	Agitation Time (Minutes)	Predicted FA ± SD	Actual FA ± SD
1	2	0.2	4	5	81.03 ± 1.27	82.06 ± 0.04
2	2	1.0	4	5	91.77 ± 0.04	92.65 ± 0.00
3	2	0.2	10	5	74.41 ± 0.04	75.53 ± 0.00
4	2	1.0	10	5	80.94 ± 0.05	80.69 ± 0.00
5	1	0.6	7	3	73.81 ± 0.09	74.85 ± 0.01
6	3	0.6	7	3	77.80 ± 0.09	78.22 ± 0.00
7	1	0.6	7	7	76.60 ± 0.01	76.41 ± 0.00
8	3	0.6	7	7	76.86 ± 0.04	77.07 ± 0.00
9	2	0.2	7	3	75.14 ± 0.18	74.69 ± 0.01
10	2	1.0	7	3	84.29 ± 0.33	84.38 ± 0.01
11	2	0.2	7	7	76.28 ± 0.22	76.17 ± 0.01
12	2	1.0	7	7	85.19 ± 0.17	85.70 ± 0.01
13	1	0.6	4	5	79.93 ± 0.79	80.36 ± 0.00
14	1	0.6	10	5	72.75 ± 0.02	73.21 ± 0.00
15	3	0.6	4	5	83.03 ± 0.06	82.41 ± 0.00
16	3	0.6	10	5	74.33 ± 0.08	74.28 ± 0.01
17	1	0.6	7	5	74.36 ± 0.37	73.29 ± 0.01
18	1	0.2	7	5	83.22 ± 0.50	83.31 ± 0.00
19	3	1.0	7	5	76.57 ± 0.08	75.59 ± 0.00
20	3	0.2	7	5	85.37 ± 0.62	85.37 ± 0.01
21	2	1.0	4	3	81.78 ± 0.24	80.52 ± 0.01
22	2	0.6	10	3	73.12 ± 0.11	72.55 ± 0.01
23	2	0.6	4	7	82.29 ± 0.15	81.18 ± 0.01
24	2	0.6	10	7	74.24 ± 0.18	73.62 ±0.01
25	2	0.6	7	5	75.85 ± 0.13	75.18 ± 0.00
26	2	0.6	7	5	75.89 ± 0.08	76.66 ± 0.00
27	2	0.6	7	5	75.92 ± 0.03	75.59 ± 0.00

**Table 3:** Removal efficiency of the flocculants.

Flocculant	COD Removal (%)	Turbidity Reduction (%)
PAC	85.66 ^a^	56.93 ^a^
Aluminium sulfate	87.86 ^a^	60.94 ^a^
Kp1	90.86 ^a,b^	92.65 ^b^
Mg1	93.12 ^b^	92.74 ^b^

Note: All measurements are mean values of triplicate experiments, with variations within 3% error limit. Alphabets (a and b) signify statistical significance (*p < 0.05*).

**Table 4:** Zeta potentials of different samples.

Sample	Zeta Potential (mV)	
	Bioflocculant Kp1	Bioflocculant Mg1
Bioflocculant solution	11.90 ± 0.87	−0.3 ± 2.44
Bioflocculant—CaCl_2_	22.93 ± 1.19	16.9 ± 0.6
Wastewater	−25.03 ± 1.36	−25.03 ± 1.36
Floc	−1.89 ± 0.74	−8.03 ± 0.31

## Data Availability

The original contributions presented in this study are included in the article/Supplementary Material. Further inquiries can be directed to the corresponding author.

## Supplementary Materials

The following supporting information can be downloaded at: https://www.mdpi.com/article/10.3390/polym17233155/s1, Table S1. Decoded factors used for optimisation of conditions of both Mg1 and Kp1 by BBD. Table S2. Coded factors used for optimisation of conditions of both Mg1 and Kp1 by BBD. Table S3. Analysis of variance (ANOVA) results for Kp1. Table S4. Analysis of variance (ANOVA) results for bioflocculant Mg1.

## Abbreviations

The following abbreviations are used in this manuscript:

OD	optical density
FA	Flocculating activity
RSM	Response Surface Methodology
FTIR	Fourier-transform infrared spectroscopy
ANOVA	Analysis of variance
SEM	Scanning electron microscopy
RE	Removal efficiency
TGA	Thermogravimetric analysis
EDX	X-ray spectroscopy
BBD	Box Behnken Design
PAC	Polyaluminium chloride
