# Supplementary material for: Characterisation, Flocculation Efficiencies and Mechanisms of Bioflocculants Derived from Klebsiella pneumoniae and Meyerozyma guilliermondii
Source: Polymers (Basel). 2025 Nov 27;17(23):3155. doi: 10.3390/polym17233155 (PMC12693871; doi:10.3390/polym17233155)
Supplement: Supplementary file 1 [file polymers-17-03155-s001.zip › polymers-3936914-supplementary.pdf]

**Supplemetary Material_polymers 3936914**

**Table S1.** Decoded factors used for optimisation of conditions of both Mg1 and Kp1 by BBD.

| **Experimental run** | **Independent variable** | | | |
|---|---|---|---|---|
| | **Cation ($CaCl_2$) (%)** | **Dosage (mg/mL)** | **pH** | **Agitation time (minutes)** |
| 1 | 2 | 0.2 | 4 | 5 |
| 2 | 2 | 1.0 | 4 | 5 |
| 3 | 2 | 0.2 | 10 | 5 |
| 4 | 2 | 1.0 | 10 | 5 |
| 5 | 1 | 0.6 | 7 | 3 |
| 6 | 3 | 0.6 | 7 | 3 |
| 7 | 1 | 0.6 | 7 | 7 |
| 8 | 3 | 0.6 | 7 | 7 |
| 9 | 2 | 0.2 | 7 | 3 |
| 10 | 2 | 1.0 | 7 | 3 |
| 11 | 2 | 0.2 | 7 | 7 |
| 12 | 2 | 1.0 | 7 | 7 |
| 13 | 1 | 0.6 | 4 | 5 |
| 14 | 1 | 0.6 | 10 | 5 |
| 15 | 3 | 0.6 | 4 | 5 |
| 16 | 3 | 0.6 | 10 | 5 |
| 17 | 1 | 0.6 | 7 | 5 |
| 18 | 1 | 0.2 | 7 | 5 |
| 19 | 3 | 1.0 | 7 | 5 |
| 20 | 3 | 0.2 | 7 | 5 |
| 21 | 2 | 1.0 | 4 | 3 |
| 22 | 2 | 0.6 | 10 | 3 |
| 23 | 2 | 0.6 | 4 | 7 |
| 24 | 2 | 0.6 | 10 | 7 |
| 25 | 2 | 0.6 | 7 | 5 |
| 26 | 2 | 0.6 | 7 | 5 |
| 27 | 2 | 0.6 | 7 | 5 |

**Table S2.** Coded factors used for optimisation of conditions of both Mg1 and Kp1 by BBD.

| Run | Cation ($CaCl_2$) (%) | Dosage (mg/mL) | pH | Agitation time (minutes) |
|---|---|---|---|---|
| 1 | –1 | –1 | 0 | 0 |
| 2 | 1 | –1 | 0 | 0 |
| 3 | –1 | 1 | 0 | 0 |
| 4 | 1 | 1 | 0 | 0 |
| 5 | 0 | 0 | –1 | –1 |
| 6 | 0 | 0 | 1 | –1 |
| 7 | 0 | 0 | –1 | 1 |
| 8 | 0 | 0 | 1 | 1 |
| 9 | –1 | 0 | 0 | –1 |
| 10 | 1 | 0 | 0 | –1 |
| 11 | –1 | 0 | 0 | 1 |
| 12 | 1 | 0 | 0 | 1 |
| 13 | 0 | –1 | –1 | 0 |
| 14 | 0 | 1 | –1 | 0 |
| 15 | 0 | –1 | 1 | 0 |
| 16 | 0 | 1 | 1 | 0 |
| 17 | –1 | 0 | –1 | 0 |
| 18 | 1 | 0 | –1 | 0 |
| 19 | –1 | 0 | 1 | 0 |
| 20 | 1 | 0 | 1 | 0 |

| | | | | |
|---|---|---|---|---|
| 21 | 0 | –1 | 0 | –1 |
| 22 | 0 | 1 | 0 | –1 |
| 23 | 0 | –1 | 0 | 1 |
| 24 | 0 | 1 | 0 | 1 |
| 25 | 0 | 0 | 0 | 0 |
| 26 | 0 | 0 | 0 | 0 |
| 27 | 0 | 0 | 0 | 0 |

**Table S3**. Analysis of variance (ANOVA) results for Kp1.

| Source | DF | Adj SS | Adj MS | F-Value | *p*-Value |
|---|---|---|---|---|---|
| Model | 14 | 639.960 | 45.711 | 35.68 | 0.000 |
| Linear | 4 | 540.920 | 135.230 | 105.56 | 0.000 |
| Dosage (mg/mL) | 1 | 221.278 | 221.278 | 172.72 | 0.000 |
| pH | 1 | 304.013 | 304.013 | 237.30 | 0.000 |
| $Cacl_2$ (%) | 1 | 10.120 | 10.120 | 7.90 | 0.016 |
| Agitation time (minutes) | 1 | 5.508 | 5.508 | 4.30 | 0.060 |
| Square | 4 | 79.957 | 19.989 | 15.60 | 0.000 |
| Dosage (mg/mL)* Dosage (mg/mL) | 1 | 35.340 | 35.340 | 27.59 | 0.000 |
| pH* pH | 1 | 17.272 | 17.272 | 13.48 | 0.003 |
| $Cacl_2$ (%)*$Cacl_2$ (%) | 1 | 0.579 | 0.579 | 0.45 | 0.514 |
| Agitation time (minutes)*Agitation time (minutes) | 1 | 0.688 | 0.688 | 0.54 | 0.478 |
| 2-Way Interaction | 6 | 19.083 | 3.180 | 2.48 | 0.085 |
| Dosage (mg/mL)* pH | 1 | 8.851 | 8.851 | 6.91 | 0.022 |

| | | | | | |
|---|---|---|---|---|---|
| Dosage (mg/mL)* $CaCl_2$ (%) | 1 | 0.096 | 0.096 | 0.08 | 0.789 |
| Dosage (mg/mL)*Agitation time (minutes) | 1 | 0.160 | 0.160 | 0.12 | 0.730 |
| pH*$CaCl_2$ (%) | 1 | 0.106 | 0.106 | 0.08 | 0.779 |
| pH*Agitation time (minutes) | 1 | 9.860 | 9.860 | 7.70 | 0.017 |
| $CaCl_2$ (%)*Agitation time (minutes) | 1 | 0.011 | 0.011 | 0.01 | 0.928 |
| Error | 12 | 15.374 | 1.281 | | |
| Lack-of-Fit | 10 | 14.214 | 1.421 | 2.45 | 0.324 |
| Pure Error | 2 | 1.159 | 0.580 | | |
| Total | 26 | 655.334 | | | |
| $R^2$ | 0.9765 | | | | |
| $R^2$-adjusted | 0.9492 | | | | |
| $R^2$-adjusted (predicted) | 0.8711 | | | | |

The DF, Adj SS, Adj MS denote degree of freedom, adjusted sum of squares, and adjusted mean squares, respectively.

**Table S4**. Analysis of variance (ANOVA) results for bioflocculant Mg1.

| **Source** | **DF** | **Adj SS** | **Adj MS** | **F-Value** | ***p*-Value** |
|---|---|---|---|---|---|
| Model | 14 | 589.225 | 42.087 | 29.13 | 0.000 |
| Linear | 4 | 465.908 | 116.477 | 80.62 | 0.000 |
| Dosage (mg/mL) | 1 | 246.613 | 246.613 | 170.69 | 0.000 |
| pH | 1 | 202.952 | 202.952 | 140.47 | 0.000 |
| $CaCl_2$ (%) | 1 | 13.462 | 13.462 | 9.32 | 0.010 |
| Agitation time (minutes) | 1 | 2.881 | 2.881 | 1.99 | 0.183 |
| Square | 4 | 113.041 | 28.260 | 19.56 | 0.000 |

| | | | | | |
|---|---|---|---|---|---|
| Dosage (mg/mL)* Dosage (mg/mL) | 1 | 93.484 | 93.484 | 64.70 | 0.000 |
| pH*pH | 1 | 15.330 | 15.330 | 10.61 | 0.007 |
| $Cacl_2$ (%)*$Cacl_2$ (%) | 1 | 0.005 | 0.005 | 0.00 | 0.954 |
| Agitation time (minutes)*Agitation time (minutes) | 1 | 0.345 | 0.345 | 0.24 | 0.634 |
| 2-Way Interaction | 6 | 10.275 | 1.712 | 1.19 | 0.376 |
| Dosage (mg/mL)* pH | 1 | 6.452 | 6.452 | 4.47 | 0.056 |
| Dosage (mg/mL)* $Cacl_2$ (%) | 1 | 0.013 | 0.013 | 0.01 | 0.925 |
| Dosage (mg/mL)*Agitation time (minutes) | 1 | 0.038 | 0.038 | 0.03 | 0.874 |
| pH*$Cacl_2$ (%) | 1 | 0.216 | 0.216 | 0.15 | 0.706 |
| pH*Agitation time (minutes) | 1 | 0.040 | 0.040 | 0.03 | 0.871 |
| $Cacl_2$ (%)*Agitation time (minutes) | 1 | 3.516 | 3.516 | 2.43 | 0.145 |
| Error | 12 | 17.338 | 1.445 | | |
| Lack-of-Fit | 10 | 15.785 | 1.579 | 2.03 | 0.374 |
| Pure Error | 2 | 1.552 | 0.776 | | |
| Total | 26 | 606.562 | | | |
| $R^2$ | 0.9714 | | | | |
| $R^2$-adjusted | 0.9381 | | | | |
| $R^2$-adjusted (predicted) | 0.8443 | | | | |

The DF, Adj SS, Adj MS denote degree of freedom, adjusted sum of squares, and adjusted mean squares, respectively.
